# Absence of renal cortical anisotropic backscattering artifact in feline chronic kidney disease

**DOI:** 10.1080/01652176.2021.1941397

**Published:** 2021-06-28

**Authors:** Ping-Hsien Chou, Hock Gan Heng, Fang-Ju Lin, Kuan-Sheng Chen

**Affiliations:** a Department of Veterinary Medicine, College of Veterinary Medicine, National Chung Hsing University, Taichung, Taiwan; b Department of Veterinary Clinical Sciences, College of Veterinary Medicine, Purdue University, West Lafayette, IN, USA; c Veterinary Medical Teaching Hospital, College of Veterinary Medicine, National Chung Hsing University, Taichung, Taiwan

**Keywords:** Cat, feline, kidney, chronic kidney disease, CKD, CABA

## Abstract

Renal cortical anisotropy backscattering artifact (CABA) is a focal hyperechoic region where the tubules are parallel to the incident ultrasound beam, reflecting most of the beams to the transducer. To investigate the association between chronic kidney disease (CKD) and the absence of renal CABA in cats. Ultrasonographic renal images of 40 cats with CKD (stage II-IV) and 36 clinically healthy cats were blindly evaluated by two observers to determine the visibility of renal CABA. Inter- and intraobserver agreements were evaluated using McNemar’s test. The association between the absence of renal CABA and CKD was assessed using Fisher’s exact test. Excellent intraobserver and substantial interobserver agreements were demonstrated. A significant association (*P* < .0001) between absent renal CABA and CKD stage was revealed in all cats. Cats with CKD had an increased risk of the absence of renal CABA (Odds ratio, 56.0; 95% CI, 13.8–227.0) compared with the clinically healthy cats. The absence of renal CABA revealed 87.5% sensitivity and 88.9% specificity to detect CKD in all cats, and 91.7% sensitivity and 83.3% specificity in aged cats. Our study demonstrated a correlation between feline CKD and the absence of renal CABA, providing a feasible and alternative method for feline CKD evaluation.

## 1. Introduction

Chronic kidney disease (CKD) is defined as an irreversible functional loss or structural abnormality of one or both kidneys, which have persisted for a prolonged period of time, typically more than 3 months (O'Neill et al. 2013). Chronic kidney disease in cats is more common in the elderly population and the prevalence increases with age; it is as high as 80.9% in cats over 10 years old (Marino et al. 2014). Serum creatinine concentration (SCrC) is most often used to analyze renal function because glomerular filtration rate is not easily measured in the clinical setting (Polzin 2013). Ultrasonography is a non-invasive technique for evaluating structural and echogenicity changes in patients with suspected CKD. This may be useful for those that potentially fulfill the criteria of CKD but do not show clinical signs (Jepson 2016).

Increased cortical echogenicity is regarded as one of the most common features of kidney disease in veterinary medicine, and an *ex vivo* study found a linear correlation between renal echogenicity and degenerative changes in cats (Zotti et al. 2015). Subjective assessment is considered unreliable because renal cortical echogenicity can be influenced by many factors such as fat accumulation in the renal cortex, ultrasound equipment setting and patient body thickness (Drost et al. 2000). Echogenicity of the liver and spleen is commonly used as an internal standard in comparison with that of the kidney in dogs and cats (Drost et al. 2000; Lee et al. 2005; Ivancic and Mai 2008; Yabuki et al. 2008; Sayre and Spaulding 2014). However, this comparison is unreliable because it is based on the assumption of a clinically normal liver or spleen (Banzato et al. 2016).

In addition to the challenges described above, the potential of renal morphology to influence the angle between the incident ultrasound beam and radial orientation of renal tubules should be considered. The cortical renal tubules that orient perpendicular to the incident ultrasound beams generate higher echogenicity as most of the beams are reflected back to the transducer. In contrast, the echogenicity is lower in the region where the renal tubules are parallel to the incident ultrasound beams (Rubin et al. 1988; Ruth et al. 2013). This is the effect of anisotropy and this phenomenon has also been observed in cats (Yabuki et al. 2008). The inclusion angles of an anisotropic effect are approximately 35° and 20° in ovine (Rubin et al. 1988) and canine (Ruth et al. 2013) kidneys, respectively. The effect does not exist in the medulla because the tubules in the medulla are tightly packed and tend to be thinner than those in the cortex, unable to produce a significant reflection (Rubin et al. 1988). Therefore, we hypothesize that focal cortical hyperechogenicity due to the backscattering artifact would be absent as the general cortical echogenicity increases in cats with CKD. The purpose of this study was to investigate the clinical correlation between the absence of the focal renal cortical anisotropic backscattering artifact (CABA) and clinicopathological staging in cats with CKD, and to evaluate whether the absence of renal CABA could be an alternative method for assessment of CKD.

## 2. Materials and methods

Medical records from the Veterinary Medical Teaching Hospital at National Chung Hsing University (NCHU) were reviewed retrospectively to retrieve cases that were clinically diagnosed with feline CKD from 2012 to 2020. A keyword ‘CKD’ within the species ‘cat’ were used to search case files. Because SCrC < 140 μmol/l was considered to be clinically normal in cats (International Renal Interest Society. , 2019), the inclusion criteria were subjects with a SCrC > 140 μmol/l. Creatinine values were measured at least twice with an interval of less than 30 days between each blood test. The CKD was considered stable when the variation in SCrC was within 25%. Renal ultrasonography was performed within the period of the SCrC testing and included at least two planes of still images (longitudinal and transverse) or cine loop. When focal hyperechogenicity could be identified in either the longitudinal or transverse plane of the kidney, where the insonation angle was relatively perpendicular to the kidney, the kidney would be regarded as having renal CABA. Subjects with renal structural abnormalities including hydronephrosis, renal or systemic neoplasia, polycystic kidney disease, or a solitary cyst diameter greater than 5 mm were excluded. Subjects with systemic diseases such as hyperthyroidism or diabetes mellitus were also excluded. The severity of feline CKD in the present study was determined according to the CKD guidelines established by the International Renal Interest Society (IRIS), and subjects with CKD stages II, III, and IV were included in the study.

The control group consisted of 36 clinically healthy subjects recruited from veterinary students and colleagues with consent, and this recruitment was approved by the Institutional Animal Care and Use Committee (approval No. 105-065 & 109-051) at NCHU. Clinically healthy subjects were confirmed by physical examination, blood pressure measurement, hematocrit measurement, serum chemistry examination, ultrasonography of urinary system, and urine urinalysis.

All ultrasound images were acquired by use of a 7.2–14 MHz linear transducer^1^ to a commercial ultrasound machine.^2^ Each kidney was imaged in at least two different planes and the images were captured and saved as Digital Imaging and Communications in Medicine files.

**Figure 1.**
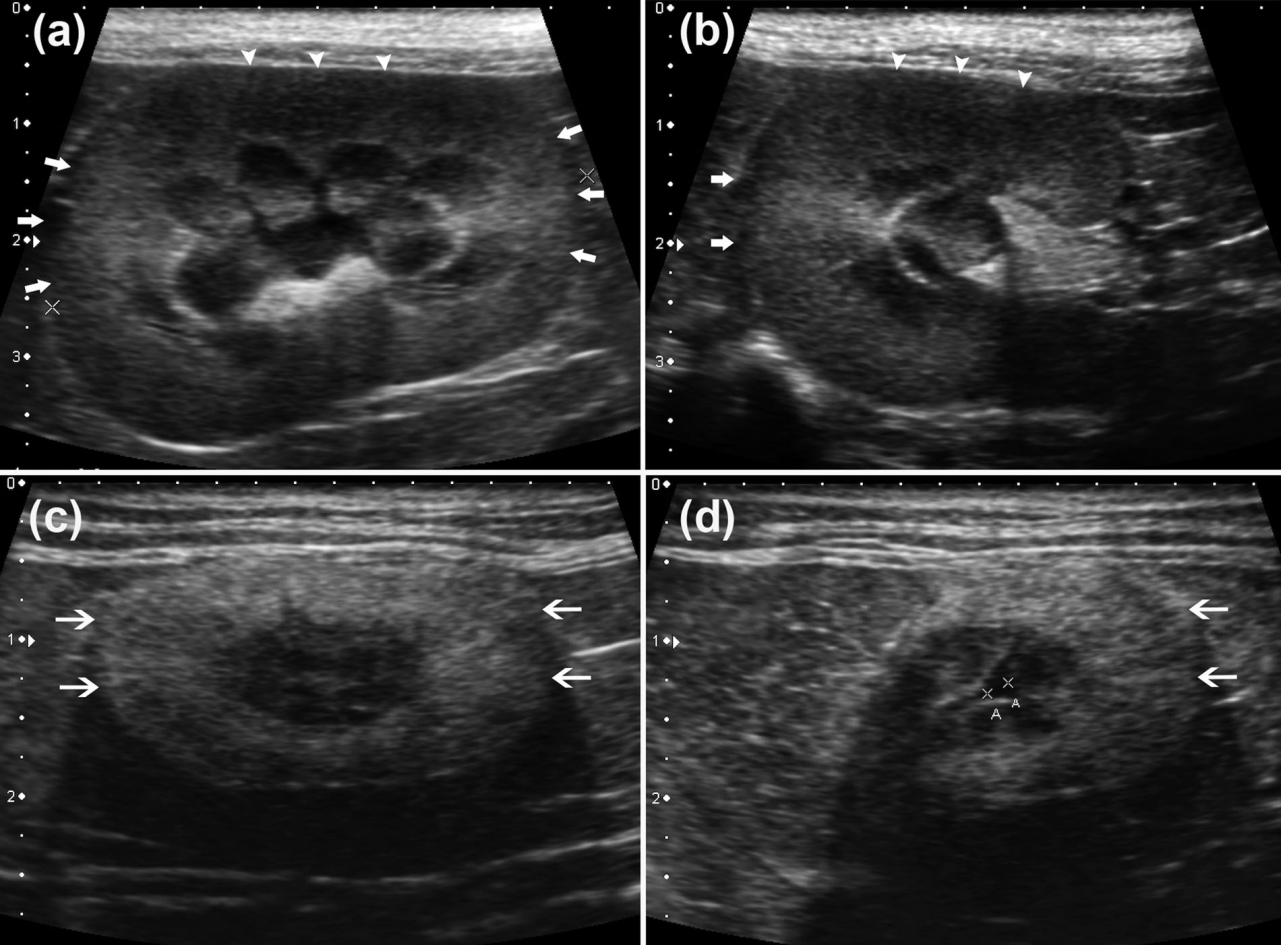
Images of the presence (a, b) and absence (c, d) of renal CABA from the control and disease groups, respectively. The focal hyperechogenic regions (brighter areas) are located approximately 3 and 9 o’clock positions (arrows) of the normal renal cortex in the longitudinal plane (a), and 9 o’clock position (arrows) in the transverse plane (b) compared with the relative hypoechoic regions (darker area) at approximately 12 o’clock position (arrowheads) in both views. Median longitudinal (c) and transverse plane (d) of a feline kidney in the disease group. The normally observed anisotropic hyperechogenicity is absent in the renal cortical region (thin arrows). The A calipers indicate mild pyelectasia.

Greyscale ultrasound images of the kidneys were reviewed using a medical image viewer software program^3^ and then evaluated in at least two planes by two observers. Both observers did not perform the ultrasound examinations and were blinded to each patient’s condition. One was a radiologist [KSC; BVM, MVS (Veterinary diagnostic imaging), PhD, MANZCVS (Veterinary Radiology)] and the other one was a radiology graduate student (PHC) with 15 years and 2 years of experience in ultrasonography, respectively. The student was trained for 30 minutes to be familiar with the ultrasonographic characteristics of renal CABA. Renal cortical regions where the incident ultrasound beams were approximately perpendicular to the renal tubules (approximately 3 and 9 o’clock positions) were compared with those parallel to the renal tubules (approximately 12 o’clock position). Focal hyperechogenicity at approximately 3 and 9 o’clock positions due to the CABA on the renal cortex was subjectively classified as ‘present’ (Figure 1a, b) and no visible focal hyperechogenicity at approximately 3 and 9 o’clock positions were classified as ‘absent’ (Figure 1c, d). The subject was included in the absence of renal CABA group when the renal CABA was absent in one or both kidneys. Both observers evaluated all the images twice within a 3-day interval.

Subjects between 7 to 16 years were included in to an ‘aged’ subgroup. Data analysis was performed on all included subjects and aged subjects using commercially available software.^4^ McNemar’s test and Cohen’s Kappa were performed to validate both the inter- and intraobserver agreements. Differences of age and body weight (BW) between control and disease groups were evaluated using Wilcoxon signed-rank test. Fisher’s exact test was used to evaluate the difference of sex and reproductive status (intact or neutered) between control and disease group. Sensitivity and specificity of the absence of renal CABA for detecting CKD were calculated as true positives/(true positives + false negatives) and true negatives/(false positives + true negatives), respectively. Fisher’s exact test was performed to determine the association between the absence of renal CABA and CKD. The increased frequency of the absence of renal CABA with the classification of CKD stages was checked by the Cochran–Armitage trend test. To investigate the associations among the variables of sex, reproductive status, BW, age and SCrC to the absence of renal CABA, univariable or multiple logistic regression was used. Statistical significance was set at *P* < .05.

## 3. Results

A total of 76 subjects (disease group *n* = 40; control group *n* = 36) were enrolled in this study, including domestic shorthair cats (*n* = 52), Chinchilla (*n* = 9), Persian (*n* = 6), American shorthair (*n* = 4), Himalayan (*n* = 2), and one each of British shorthair, Siamese, and Norwegian Forest. Forty-two out of 76 subjects were aged subjects (disease group *n* = 24; control group *n* = 18).

**Figure 2.**
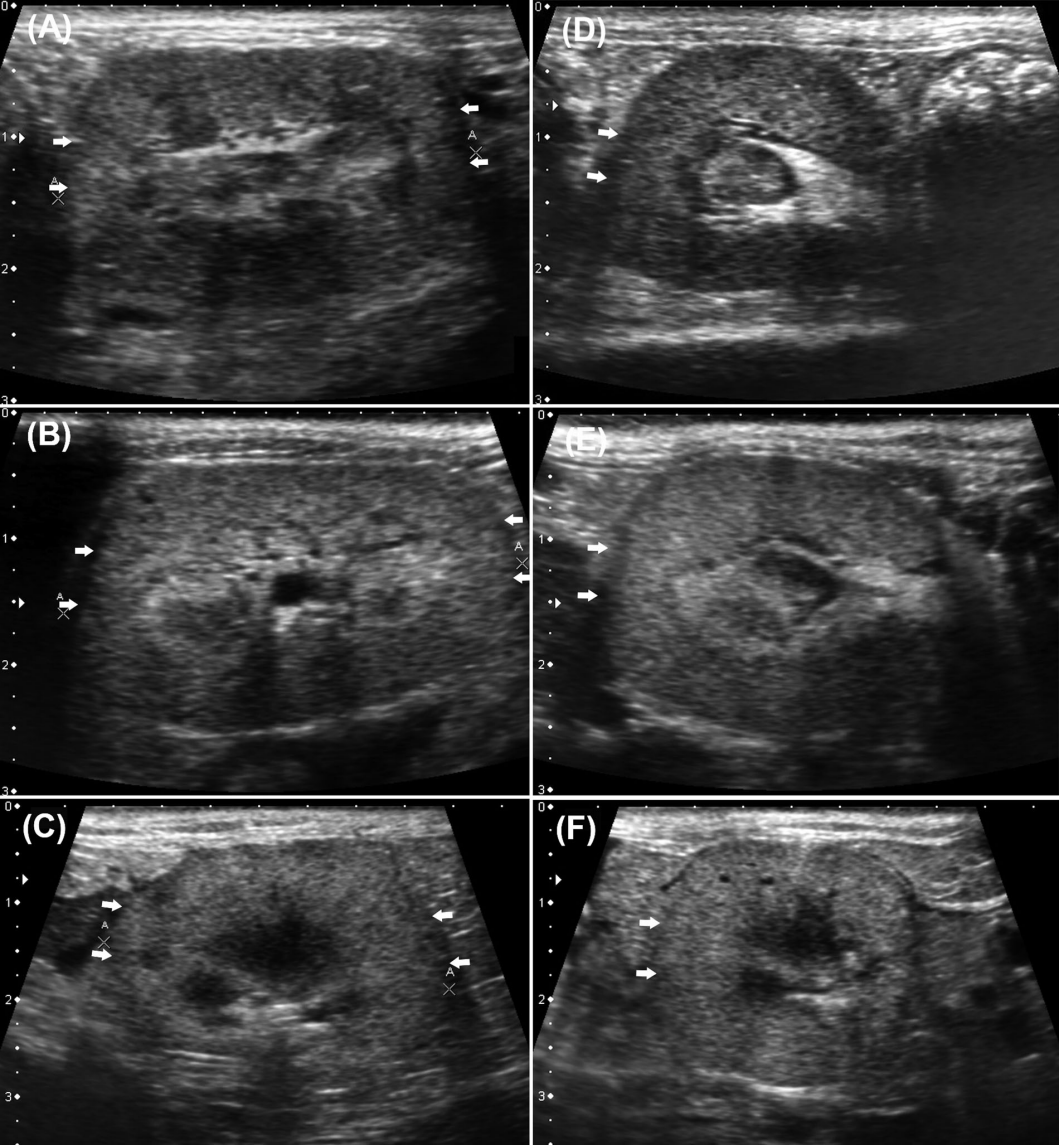
Images of ultrasound images containing longitudinal (a-c) and transverse (d-f) planes in the renal cortex in CKD stage II (a, d), CKD stage III (b, e), and CKD stage IV (c, f) cats. No CABA is observed in the renal cortical regions (arrows) in all the images.

An illustration of ultrasonography of each CKD stage in the disease group is shown in Figure 2. Intraobserver evaluation of the absence of renal CABA showed agreement (*P* = .56, McNemar test), and had a Cohen’s Kappa value of .81, indicating excellent (> .80) agreement (Landis and Koch 1977). Interobserver evaluation of the absence of renal CABA was in agreement (*P* = 1.0, McNemar test), and the Cohen’s Kappa value was .73, defined as substantial (.61– .80) agreement (Landis and Koch 1977). The radiologist’s results were chosen for further analyses in this study.

In all the included subjects, age (*P* < .0001) and BW (*P* = .0009) differed significantly between control and disease groups (Table 1). Sex distribution (*P* = .06) and reproductive status (*P* = .07) were not significantly associated with the absence of renal CABA (Table 1). A significant association (*P* < .0001; odds ratio, 56.0; 95% CI, 13.8–227.0) was found between the absence of renal CABA and CKD stage, and the Cochran–Armitage trend test also indicated a significant classification of CKD stage (*P* < .0001; Table 2). The absence of renal CABA showed an 87.5% sensitivity and 88.9% specificity of detecting CKD. Regarding the detection of the different CKD stages, the sensitivity was 76.5%, 92.3%, and 100% for stage CKD II, III, and IV, respectively (Table 2). Univariable logistic regression analysis showed sex (*P* = .01), age (*P* = .0008) and SCrC (*P* = .0001) were significantly associated with the absence of renal CABA (Table 3). On multivariable analysis, only age and SCrC were significantly associated the absence or renal CABA.

**Table 1.** Demographic statistics of all included cats and subgrouped aged cats (7–16 years old).

	Total number of cats (n = 76)	All cats Control group (n = 36)	Disease group (n = 40)	*p*
Age				
mean ± SD	9.7 ± 5.04	–	–	–
Median (range)	–	7 (2–14)	13.5 (2–20)	< **.0001**
BW (kg)				
Median (range)	4 (2.1–8.7)	4.9 (2.8–8.7)	3.6 (2.1–7)	**.0009**
Sex				
Male	43	16	27	
Female	33	20	13	.06
Reproductive status				
Intact	13	3	10	
Neutered	63	33	30	.07
	Subgrouped aged cats (7–16 years old)			
	Total number of cats (n = 42)	Control group (n = 18)	Disease group (n = 24)	***p***
Age				
Median (range)	11 (7–16)	10 (8–14)	12.5 (7–16)	.07
BW (kg)				
Median (range)	4 (2.1–8.7)	4.9 (2.8–8.7)	3.5 (2.1–6.5)	**.02**
Sex				
Male	24	7	17	
Female	18	11	7	.06
Reproductive status				
Intact	7	1	6	
Neutered	35	17	18	.21

Age and BW were analyzed by Wilcoxon signed-rank test; sex and reproductive status were analyzed by Fisher’s exact test. A statistical significance (*P* < .05, in bold) exists between the groups. SD, standard deviation. BW, body weight.

**Table 2.** Presence or absence of renal CABA in different CKD stages in all cats and subgrouped aged cats (7–16 years old).

			All cats CABA			
			Presence	Absence	*P*^＃^	*P*^†^
Control			32/36 (88.9%)	4/36 (11.1%)	< .0001*	< .0001*
Disease		CKD stage II	4/17 (23.5%)	13/17 (76.5%)		
		CKD stage III	1/13 (7.7%)	12/13 (92.3%)		
		CKD stage IV	0/10 (0%)	10/10 (100%)		
Total number			*n* = 37	*n* = 39	76	
			Subgrouped aged cats (7–16 years old) CABA			
			Presence	Absence	***P***^＃^	***P***^†^
Control			15/18 (83.3%)	3/18 (16.7%)	< .0001^＃^	< .0001^†^
Disease	CKD stage II		2/10 (20%)	8/10 (80%)		
	CKD stage III		0/9 (0%)	9/9 (100%)		
	CKD stage IV		0/5 (0%)	5/5 (100%)		
Total number			*n* = 17	*n* = 25	42	

＃ A significant association exists between feline CKD groups (including stage II, III, and IV) and the absence of renal CABA is determined by Fisher’s exact test.

† A significantly increased frequency of the absence of renal CABA with the classification of CKD stages is determined by Cochran–Armitage trend test.

Percentages represent the distribution of the presence and absence of focal hyperechogenicity on the renal cortex in each group and CKD stage. CABA, cortical anisotropic backscattering artifact. Statistical significance *P* < .05

**Table 3.** Univariable and multivariable logistic regression analysis for sex, reproductive status, BW, age and SCrC associated with the absence of renal CABA.

	All cats					
Univariable analysis				Multivariable analysis		
	*P*	OR	95% CI	*P*	OR	95% CI
Sex	**.01**	.27	.10–.70	.12	.30	.07–1.37
RS	.16	.40	.11–1.45			
BW	.22	.82	.60–1.12	–	–	–
Age	**.0008**	1.20	1.08–1.34	**.04**	1.18	1.01–1.38
SCrC	**.0001**	13.77	3.58–52.90	**.002**	7.82	2.17–28.14
Subgrouped aged cats (7–16 years old)						
Univariable analysis				Multivariable analysis		
	*P*	OR	95% CI	*P*	OR	95% CI
Sex	**.02**	.21	.06–.80	.58	.59	.09–3.93
RS	.15	.20	.02–1.82			
BW	.49	.88	.61–1.26	–	–	–
Age	.38	1.11	.88–1.41	**–**	**–**	**–**
SCrC	**.006**	27.17	2.58–286.40	**.007**	25.92	2.43–276.54

Data in bold are statistical significance (*P* < .05). RS, reproductive status. BW, body weight. SCrC, serum creatinine concentration. OR, odds ratio. CI, confidence interval.

In the subgroup of aged subjects (7–16 years old), significant differences were only observed in BW (*P* = .02, Table 1). Sex distribution (*P* = .06) and reproductive status (*P* = .21) were also not significantly associated with the absence of renal CABA (Table 1). Significant associations (*P* < .0001; odds ratio, 55.0; CI, 8.18–369.9) were found between the absence of renal CABA and CKD stage, and the Cochran–Armitage trend test also indicated the significant ordering of CKD stage (*P* < .0001, Table 2). The absence of renal CABA showed 91.7% sensitivity and 83.3% specificity to detect CKD. For the detection of the different CKD stages, the sensitivity was 80.0%, 100%, and 100% for stage CKD II, III and IV, respectively (Table 2). Univarible logistic regression analysis showed that sex (*P* = .02) and SCrC (*P* = .006) were significantly associated with the absence of renal CABA, while only the SCrC (*P* = .007) was significantly associated with the absence of renal CABA on multivariable analysis (Table 3).

## 4. Discussion

The findings of this study confirmed the association between feline CKD and the absence of renal CABA. A positive correlation was observed between the absence of renal CABA and the severity of CKD. Common histopathological lesions in CKD, such as tubular degeneration, interstitial nephritis, fibrosis, and glomerulosclerosis, have been shown to be significantly more severe in advanced CKD stages than in the early stages (McLeland et al. 2015). In addition, both degenerative and inflammatory lesions in cats exhibited a positive correlation between cortical echogenicity and lesion severity score (Zotti et al. 2015). Since cats with CKD have generalized increased renal cortical echogenicity caused by the histopathological lesions (Zotti et al. 2015), the absence of renal CABA is most likely associated with these cortical histopathological lesions that masks or eliminates CABA. Thus, presence or absence of renal CABA may be used as an alternative method of evaluation of renal cortical echogenicity without comparing to other organs such as the liver or spleen as recommended previously (Yabuki et al. 2008; Sayre and Spaulding 2014).

This study also illustrated that the absence of renal CABA was positively associated with the CKD stages in the IRIS system. The more severe the CKD stages, the higher sensitivity of detection of CKD with the absence of renal CABA. This ultrasonographic finding has not been described previously in cats. Recently, decreased renal cortical thickness has been reported to be associated with loss of renal function in cats (Yan et al. 2020). The combination of the absence of renal CABA and a thin renal cortex may indicate higher probability to detect higher stage of CKD in cats.

The absence of renal CABA was not influenced by sex, reproductive state, or BW in this study. Although age and SCrC were significantly associated with the absence of renal CABA in all cats, increased SCrC had much higher odds for the absence of renal CABA than increased age. Because aged cats are predisposed to CKD, we removed the young population (1–6 years old) to conduct a better age-matched evaluation for the absence of renal CABA in the aged cats. The results revealed that the absence of renal CABA was not influenced by age, but was highly associated with increased SCrC, indicating that the absence renal CABA could permit the differentiation of CKD and normal kidney in cats.

An excellent intraobserver agreement and a substantial interobserver agreement were established between the experienced radiologist and the radiology graduate student in this study. It is proposed that this diagnostic imaging evaluation may be less likely affected by observer experience. With knowing the ultrasonographic characteristics of renal CABA, this method provides a feasible method for evaluating change in renal cortical echogenicity.

In addition to CABA, true renal pathological lesions such as neoplasia, infection, mineralization, fibrosis, gas, and chronic infarcts may also cause focal increase in renal cortical echogenicity (Ruth et al. 2013). It is important to recognize renal CABA and not mistake it for other focal hyperechoic lesions in ultrasonography. A focal renal cortical infarct, normally a wedged-shaped hyperechoic area independent of the angle of insonation, may cause focal cortical indentation and acoustic shadowing in the chronic infarct (Brown et al. 1990). In contrast, renal CABA in this study did not cause acoustic shadowing as shown in a previous study (Ruth et al. 2013). Since renal CABA is attributed to gradual variability in incident ultrasound beam angle and renal tubules, CABA does not have well-defined margins but a diffuse border and transition zone between renal CABA and the adjacent renal cortex (Ruth et al. 2013). Therefore, this angle-dependent characteristic could help to distinguish CABA from true lesions.

Despite the positive correlation, not every kidney in the disease group had absent CABA; this could be explained by the linear increase in echogenicity with the severity of inflammation and degeneration. However, within the control group, renal CABA was absent in 11.1% and 16.7% subjects of all subjects and aged subjects, respectively. Several justifiable explanations are proposed. Individual variation or bias in subjective evaluation had to be considered. In addition, there might be some early occult renal diseases in the control group because the SCrC is known to not increase until the loss of renal function reaches 75% (Brown et al. 1990). Furthermore, fat accumulation in kidneys has been attributed to generalized increased renal cortical echogenicity in cats (Drost et al. 2000), that may interfere with the identification of the renal CABA. However, the correlation between the absence of renal CABA and fat accumulation in the renal cortex needs further investigation.

The major limitation of this study was lack of histological examination. We were unable to draw comparisons between the absence of renal CABA and histological changes. Small sample size with a wide 95% CI in the univariable logistic regression was another limitation and may increase the probability of type I error. Symmetric dimethylarginine is sensitive to detect CKD stage I, but was not available in our veterinary hospital until June 2018; therefore, subjects with early CKD with SCrC < 140 μmol/l may have been included in the control group.

In conclusion, a significant association and ordering between feline CKD stages and the absence of renal CABA have been identified in this study, and showed sensitivities of 87.5% and 88.9% and specificities of 91.7% and 83.3% in the detection of CKD in all and aged cats, respectively. With knowing the ultrasonographic characteristics of renal CABA, the excellent intraobserver and substantial interobserver reproducibilities make this subjective ultrasonographic evaluation reliable. In addition, absence of renal CABA was not influenced by age and BW in the aged cats. We have demonstrated this internal auto-referencing method to assess changes in renal cortical echogenicity, but the findings should be interpreted in combination with clinicopathological results during clinical ultrasonographic examination.

## Notes

1 PLT-1204 BT, Toshiba Medical Systems Corporation, Otawara, Japan.

2 Xario SSA-660A, Toshiba Medical Systems Corporation, Otawara, Japan.

3 RadiAnt DICOM viewer, version 4.2.1, Medixant, Poznan, Poland.

4 SAS, version 9.4, SAS Institute Inc, Cary, NC, USA.
